# The Risk of Subsequent Miscarriage in Pregnant Women with Prior Polycystic Ovarian Syndrome: A Nationwide Population-Based Study

**DOI:** 10.3390/ijerph18168253

**Published:** 2021-08-04

**Authors:** Mei-Lien Pan, Li-Ru Chen, Kuo-Hu Chen

**Affiliations:** 1 Institute of Information Science, Academia Sinica, Taipei 115, Taiwan; mlpan66@gmail.com; 2 Department of Physical Medicine and Rehabilitation, Mackay Memorial Hospital, Taipei 104, Taiwan; gracealex168@gmail.com; 3 Department of Mechanical Engineering, National Yang Ming Chiao Tung University, Hsinchu 300, Taiwan; 4 Department of Obstetrics and Gynecology, Taipei Tzu-Chi Hospital, Buddhist Tzu-Chi Medical Foundation, Taipei 231, Taiwan; 5 School of Medicine, Tzu-Chi University, Hualien 700, Taiwan

**Keywords:** polycystic ovarian syndrome (PCOS), miscarriage, abortion, metformin

## Abstract

***Objective***: To assess the risk of subsequent miscarriage in pregnant women with a prior diagnosis of polycystic ovarian syndrome (PCOS). ***Methods***: Using a nationwide, population-based database (Taiwan National Health Insurance Research Database) during 1998–2012, the study retrieved 1,000,000 randomly-sampled insured citizens as research subjects. The women with a diagnosis of pre-pregnancy PCOS (*n* = 13,562) who had chromosomal anomalies, artificial abortion, inconsistent diagnoses, and who were initially diagnosed with PCOS at >45 or <15 year-old were excluded, respectively. The records of gynecologic ultrasonography and/or blood tests were checked to verify the accuracy of the diagnoses of both PCOS and miscarriage (ICD-9 CM codes). After pregnancy, every woman with prior PCOS was age-matched to four women without prior PCOS. ***Results***: Pregnant women with prior PCOS (the case group; *n* = 1926) and those without prior PCOS (the control group; *n* = 7704) were compared. The incidence of subsequent miscarriage was much higher in the case group compared with the control group (33.80% vs. 4.09%, *p* < 0.0001). Logistic regression analysis revealed that the risk of subsequent miscarriage was significantly higher in the case group than the control group (odds ratio [OR] 11.98; 95% CI 10.34–13.87, *p* < 0.0001), and the result remained similar while adjusted with covariates (adjusted OR 11.97; 95% CI 10.27–13.95, *p* < 0.0001). In the case group, the patient who used metformin had a lower risk of subsequent miscarriage (adjusted OR 9.53; 95% CI 6.69–13.57) when compared with those who did not receive metformin treatment (adjusted OR 12.13; 95% CI 10.38–14.18). ***Conclusion***: For pregnant women, a pre-pregnancy diagnosis of PCOS is an independent and significant risk factor for subsequent miscarriage. The risk of subsequent miscarriage is reduced by about 1/4 for the PCOS patients who undergo metformin treatment compared with those who do not.

## 1. Introduction

**Figure 1:**
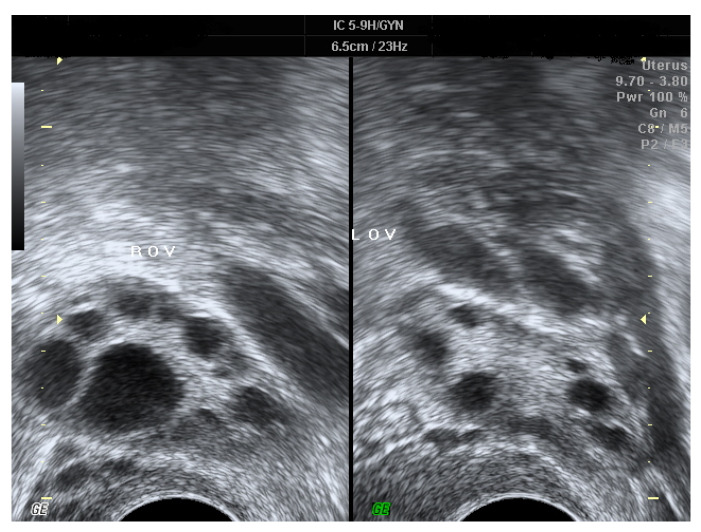
The ultrasonic images of PCOS, which are characterized by the presence of multiple small “necklace-like” follicles in both ovaries and/or increased ovarian volume.

Polycystic ovarian syndrome (PCOS) is the most common endocrine disorder of reproductive-age women [1]. According to the Rotterdam criteria [2], it refers to ovarian dysfunction presented with two of the following three features: chronic anovulation, hyperandrogenism, and special morphologic changes of bilateral ovaries [1,2,3,4,5]. Under ultrasonography, PCOS is characterized by the presence of multiple small follicles in both ovaries and/or increased ovarian volume [2,4] (Figure 1). The prevalence of PCOS is estimated to be 12%–20% among women of reproductive-age [1,4,6,7], and affected women often manifest symptoms and signs including androgen excess, obesity, infertility, and menstrual irregularity [5]. Even though the etiology of PCOS remains unclear and is deemed multi-factorial, current evidence reveals that gene-related resistance to insulin may be the fundamental cause underlying PCOS, with consequent hyperinsulinemia to stimulate excess production of ovarian androgen and to block maturation of follicles [4,8,9,10,11]. More than half of the PCOS patients have coexistent metabolic syndrome [12,13], in whom insulin resistance is a widespread finding and the probability of adult-onset diabetes mellitus is five–eight-fold compared to females without PCOS [13,14]. Additionally, several immunologic disorders, including elevated levels of cytokines [8,15] as well as autoimmune antibodies [16,17,18,19] and immune diseases [16,20], are more common amongst PCOS women. There is an association between PCOS and autoimmune diseases, such as anti-nuclear antibody (ANA) and anti-TPO that have been documented in systemic lupus erythematosus (SLE) and Hashimoto thyroiditis, respectively [21].

Miscarriage is defined as a spontaneous loss of an intrauterine pregnancy before the fetus can survive outside the uterus [22]. It occurs at less than 20 weeks’ gestation and affects up to 20 percent of recognized pregnancies [23]. Chromosomal abnormalities are the most common cause of first trimester miscarriage and are detected in 50–85% of pregnancy tissue specimens after spontaneous miscarriage [22]. Multiple other causative factors also may play a role [23]. Other less common causes of miscarriage include antiphospholipid syndrome, inherited thrombophilias (antithrombin deficiency, deficiency of protein C and protein S), and congenital structural abnormalities of the uterus. The risk of miscarriage is also increased in women with poorly controlled diabetes or disease of the thyroid gland [22].

In modern practice, pelvic ultrasonography has become the accepted standard for examining women with suspected complications of early pregnancy [22,24,25]. As mentioned above, miscarriage is usually diagnosed by routine ultrasonography or when an ultrasound scan is obtained because the symptoms and physical signs of pregnancy are regressing [23]. A pregnancy is diagnosed as miscarriage (nonviable) if it meets one of the commonly accepted positivity criteria for that diagnosis, such as the embryonic size at which nonvisualization of a heartbeat on ultrasonography is diagnostic of failed pregnancy [26].

Although research has investigated the effects of PCOS on pregnancy outcomes in women undergoing assisted reproductive technique (ART) treatment [27,28,29], fewer studies have explored the association of pre-pregnancy PCOS with subsequent miscarriage in naturally conceived pregnancies. Due to the common inflammatory disorders associated with both PCOS and miscarriage, there is a possible linkage between these two pathologies. However, most of the previous studies used physician-identified criteria for PCOS and miscarriage. In contrast to an objective standard used for the diagnosis, subjective identification of PCOS and miscarriage by physicians may lead to selection bias. Furthermore, most of the previous studies were conducted in a single hospital with either a small sample size or a special population (women undergoing ART treatment), thus the conclusions were limited and less powerful. In view of these limitations, the present study aims to evaluate the risk of subsequent miscarriage in pre-pregnancy PCOS women, by means of using stricter selection criteria to analyze a large nationwide population-based sample.

## 2. Materials and Methods

### 2.1. The Research Database and the Sample Source

Launched in 1995, the National Health Insurance (NHI) system has covered 93% of the total citizens in Taiwan in 1997, and 99% in 2010. Under the NHI system, International Classification of Diseases-9th Revision-Clinical Modification (ICD-9-CM) and the NHI-specific codes are separately entered for disease diagnoses and treatments or procedures, respectively. For research purposes, the National Health Insurance Research Database (NHIRD) was released from the claim data of the NHI program, and used in the current study for further analyses. Currently, existing data in the NHIRD have been de-linked by means of erasing the identification codes of insured citizens and health care practitioners. The nationwide population-based database consisted of outpatient and inpatient medical data of insured patients, including basic demographics, time of medical services, clinical diagnoses, and medical records of prescription. As a subset of NHIRD, Longitudinal Health Insurance Database (LHID) 2010 was particularly designed for cohort studies, and it contained a longitudinal dataset of 1,000,000 randomly sampled citizens who were insured in 2010. There were no significant differences of sex, age, or healthcare expenditure between the LHID 2010 subset and the data of total insured citizens in the NHI system. The current study did not recruit participants while obtaining their consents, but enrolled and analyzed eligible women using the anonymous database of NHIRD. As a result, no women “agreed” or “refused” to participate in the study. The major causes of data censoring were the withdrawal from the NHI system and deaths of insured citizens.

### 2.2. The Study Ethics

Since all data in the NHIRD has been de-linked and anonymized, informed consent for the current study is waived according to local regulations. The Institutional review board of Taipei TzuChi hospital, Taiwan has approved the study (No: 06-W08-060).

### 2.3. Criteria for Case Inclusion and Exclusion

Retrieved from the LHID 2010 subset, women who had a diagnosis of PCOS (ICD-9-CM code 256.4) and were aged 15–45 during 1998 and 2012 were enrolled as the case group. As the current study evaluated the risk of subsequent miscarriage (ICD-9-CM code 632; 634.X; 637.X; 646.3X) for pregnant women with pre-pregnancy PCOS, the focus group was reproductive-aged women and thus excluded PCOS women diagnosed at >45 or <15 year-old. At the same time, insured citizens with inconsistent PCOS diagnoses were also not included. For the prevention of incorrect diagnosis by wrong coding, the records of gynecologic ultrasonography and blood tests were checked to verify the accuracy of the diagnoses of PCOS. In case the diagnoses of PCOS were made without accompanying hormone tests of blood testosterone, FSH, or LH (NHI-specific codes: 09121B, 09121C, 09064B2, 09125C, 09125B, 09078B1, 09078B2, 09126B, 09126C) and pelvic ultrasonography (NHI-specific code: 19003C), the diagnoses were recognized as invalid. The results of hormone tests and the findings of gynecologic ultrasonography were reviewed to verify the PCOS diagnosis. Furthermore, pre-pregnancy chromosomal anomaly is a significant risk factor for miscarriage, and women with the disorder should be excluded in the study to avoid interference.

**Figure 2:**
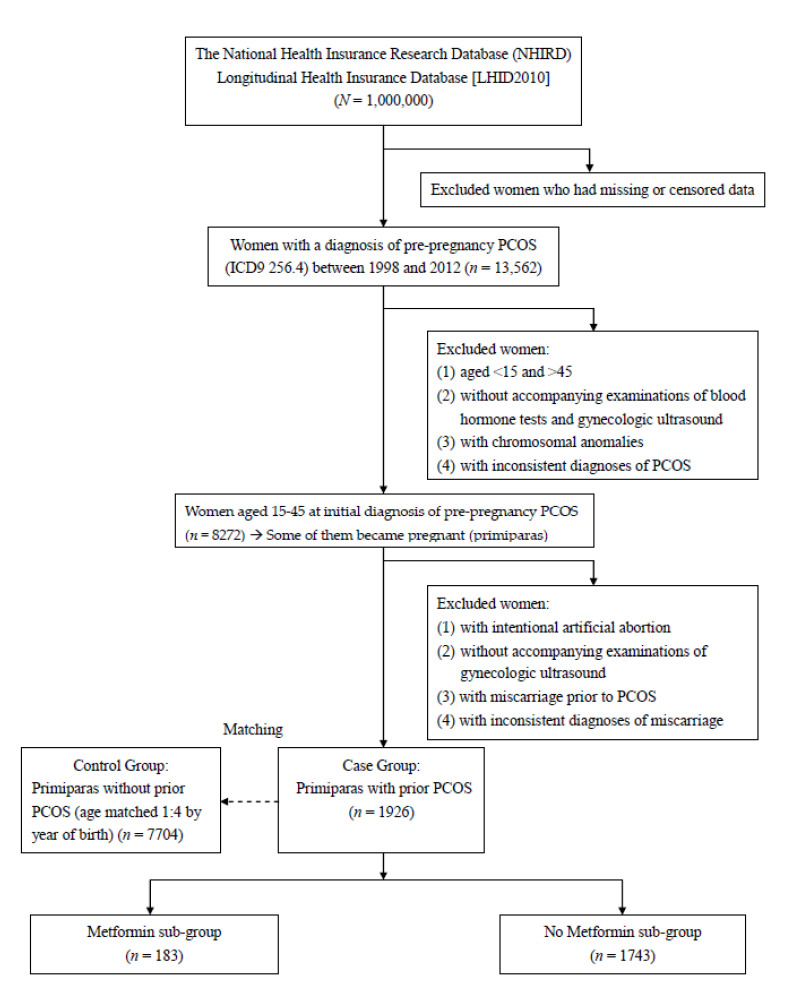
The flowchart of case inclusion, exclusion, and classification for the women with/without pre-pregnancy PCOS.

In the next stage, the PCOS women who became pregnant and experienced subsequent miscarriage were analyzed. We excluded the women who had elective termination, inconsistent diagnoses, miscarriage prior to PCOS, and who were diagnosed without accompanying gynecologic ultrasonography. For affected females, the diagnoses of miscarriage were not recognized as valid without the reports of pelvic ultrasonography. Since our focus group was pregnant women with miscarriage (spontaneous pregnancy loss), we excluded the women who underwent elective termination. At the same time, insured citizens with inconsistent miscarriage diagnoses were not included. Established on the basis of clinical manifestations, ultrasonic features and biochemistry analysis reports, the coding and diagnoses for miscarriage and PCOS are stricter and more precise. Finally, each pregnant woman (prior PCOS; the case group) was matched to four women of the same age (no prior PCOS; the control group). Figure 2 displays the flowchart of case inclusion, exclusion, and classification for the women with/without pre-pregnancy PCOS.

In addition to evaluating the risk of pre-pregnancy PCOS on subsequent miscarriage, the study further explored the effect of metformin used for treating PCOS. As a hypoglycemic drug and insulin sensitizer, metformin can reduce androgen levels and restore ovulation [11,30]. It can lower blood insulin and androgen levels to improve hyperandrogenism [11,30]. The PCOS women were subsequently classified into two sub-groups depending on metformin use (Figure 2).

The general characteristics, such as age at PCOS diagnosis, age at first pregnancy, occupation, economic status, degree of urbanization, and co-morbidities were compared for women between the case and control groups. The main environmental factors affecting PCOS included economic status, geography and occupation [31]. The demographics “occupation” was categorized as white collar, blue collar, retired, and others. The degree of urbanization was divided into urban, suburban, and rural. The economic status of insured citizens was represented by the classification of insurable wage under the NHI system (currency exchange rate: 1 US$ = 27.79 NTD), and thus classified into four levels: insurable wage ≥40,000 NTD; 20,000–40,000 NTD; < NTD 20,000; and retired/others). As possible confounding factors, co-morbidities (ICD9-CM codes) were listed as follows: diabetes mellitus (250.X); dyslipidemia (272.X); hypertension (401–405.X); cerebrovascular disease (430–438.X); ischemic heart disease (410–414.X); chronic pulmonary disease (490–496.X); and autoimmune disease. Autoimmune disease includes Sjogren’s syndrome (710.2), SLE (710.0), rheumatoid arthritis (RA) (714.0; 714.30–33), vasculitis (446.X; 443.1), Behcet’s disease (136.1), systemic sclerosis (710.1), dermatomyositis (710.3), polymyositis (710.4), pemphigus (694.4), Kawasaki disease (446.1), ulcerative colitis (556.0–6; 556.8–9), and Crohn’s disease (555.X).

### 2.4. Data Analysis

SAS^®^ version 9.4 (SAS Institute, Inc., Cary, NC, USA) was employed for the data analysis. Between the case and control groups, the general characteristics include age, demographics and co-morbidities were compared with Student’s t test and Pearson’s χ^2^, as appropriate. Logistic regression analyses were performed to estimate the odds ratio (OR) and 95% conference interval (CI) of the risks after multivariate adjustment with possible confounding factors. The level of significance was set as a *p*-value <0.05.

## 3. Results

Figure 2 illustrated the flowchart of case selection. In the present study, a total of 1,000,000 randomly-sampled insured citizens were retrieved from the database. After initial screening, there were 13,562 women with a pre-pregnancy diagnosis of PCOS eligible for a further study. The major causes of data censoring were the withdrawal of insured citizens from the NHI system and deaths of insured citizens. In the second stage, PCOS women who had chromosomal anomalies, inconsistent diagnoses, and who were initially diagnosed with PCOS at >45 or <15 year-old were excluded, respectively. At the same time, the records of gynecologic ultrasonography and blood tests were checked to verify the accuracy of the diagnoses of PCOS. In the third stage, the PCOS women (*n* = 8272) who became pregnant and aborted were analyzed by excluding those who had elective termination, inconsistent diagnoses, miscarriage prior to PCOS, and those who were diagnosed without accompanying gynecologic ultrasonography. Each pregnant woman with PCOS was age-matched to 4 women without PCOS. Overall, pregnant women with prior PCOS (the case group; *n* = 1926) and those without prior PCOS (the control group; *n* = 7704) were compared.

Table 1 showed a comparison of the characteristics of the pregnant women between the case and control groups. The item “Age at PCOS diagnosis” indicates the average age of these women when PCOS was first diagnosed; while the item “Age at first pregnancy” indicates the average age of these women when they first became pregnant. In both groups, the mean age at PCOS diagnosis was 27.31 ± 5.06 years. The age at first pregnancy was relatively older in the case group in contrast to the control group (30.03% vs. 27.33%, *p* < 0.0001). In both groups, the dominant occupation, urbanization and economic status were white collar (>55%), urban (>60%), and 20,000–40,000 NTD (>40%), respectively. Generally, there were significant differences in the items of occupation (*p* < 0.0001), degree of urbanization (*p* = 0.0005), and economic status (*p* < 0.0001) between the case and control groups (Table 1). With regard to co-morbidities, pregnant women in the case group had an elevated incidence of diabetes mellitus (*p* < 0.0001) and dyslipidemia (*p* = 0.0001) in comparison to the control group.

The risk analysis of subsequent miscarriage for pregnant women with prior PCOS is shown in Table 2. Subsequent miscarriage occurred more frequently in the case group than in the control group (33.80% vs. 4.09%, *p* < 0.0001). Logistic regression analyses demonstrated that the risk of subsequent miscarriage was much higher in the case group than in the control group (crude OR 11.98; 95% CI 10.34–13.87, *p* < 0.0001), and the result remained similar while adjusted with covariates (adjusted OR 11.97; 95% CI 10.27–13.95, *p* < 0.0001).

In the case group, the sub-group analysis is also presented in Table 2. Among 1926 pregnant women with pre-pregnancy PCOS, 183 underwent metformin treatment for PCOS. The characteristics of PCOS patients between metformin users and nonusers were compared. Although metformin usage was often indicated for patients with concurrent PCOS and diabetes mellitus, there was no difference in the distribution of diabetes mellitus between metformin users and nonusers (4.65% vs. 4.36%, *p* > 0.05). Likewise, there were no differences of the characteristics including age, occupation, urbanization, economic status, and co-morbidities (all *p* > 0.05) between metformin users and nonusers. In the case group, the patient who used metformin had a lower risk of subsequent miscarriage (adjusted OR 9.53; 95% CI 6.69–13.57) when compared with those who did not receive metformin treatment (adjusted OR 12.13; 95% CI 10.38–14.18). The risk of subsequent miscarriage was reduced by about 1/4 for the PCOS patients who underwent metformin treatment compared with those who did not.

## 4. Discussion

The result of the current study demonstrated that pregnant women with pre-pregnancy PCOS had a much higher incidence of subsequent miscarriage than those without pre-pregnancy PCOS (33.80% vs. 4.09%, *p* < 0.0001). Further analyses showed that women in Taiwan with pre-pregnancy PCOS had a >10-fold increased probability of miscarriage in comparison to those without pre-pregnancy PCOS (adjusted OR 11.97; 95% CI 10.27–13.95). Since the women with prior PCOS are at elevated risk for subsequent miscarriage, they should be offered relevant information and suggestions as to facilitate specialty referral and early management. For affected women, counselling and management can be arranged in response to potentially physical effects and psychological impacts.

Autoimmune diseases [16,20], congenital gene or chromosomal abnormalities [9,10] and decreased progesterone levels are relatively common in both patients with PCOS and with miscarriage. These patients often have coexistent autoimmune thyroiditis, SLE, RA, as well as elevated anti-nuclear, anti-ovarian, and anti-FSH antibodies [16,17,18,19,20]. Although the association with immunologic diseases has been clarified in previous research, the direct linkage between PCOS and miscarriage remains unclear. One possible and reasonable explanation for our finding is that both PCOS and miscarriage have common intrinsic immunologic factors. Under such a hypothesis, these two diseases should demonstrate an identical immunologic pathology, similar immune disorders, different other etiologies, and varying phenotypic expressions. This hypothesis is supported by several studies that investigated the nature of these two diseases. Low level of progesterone in PCOS caused overstimulation of immune system that produced more estrogen which led to various auto-antibodies, such as anti-nuclear (ANA), anti-thyroid, anti-spermatic, anti-SM, anti-histone, anti-carbonic anhydrase, anti-ovarian, and anti-islet cell antibodies [21]. Likewise, miscarriage resulted from impaired progesterone synthesis, an endocrine defect in turn associated with ovarian resistance to the gonadotropic effects of prolactin. Miscarriage also required the pro-inflammatory cytokine TNF-α and correlated with the luteal induction of the prolactin receptor signaling inhibitors suppressor of cytokine signaling. Such links between immune activation and reproductive endocrine dysfunction may be relevant to pregnancy loss [32]. Other studies revealed the significantly increased TNF-α level [15,33] and decreased membrane component 1 of the progesterone receptors [34] in patients with PCOS. Consequently, the down-regulated anti-apoptotic effect and cumulative tissue destruction may result in the clinical manifestations of these two diseases.

Previous research also supports the immunologic opinion. Many pregnancy complications including miscarriage, fetal growth restriction, gestational hypertension, and preterm birth are associated with excessive or misdirected complement activation. Clinical studies employing complement biomarkers in plasma and urine implicated dysregulated complement activation in components of each of the adverse pregnancy outcomes [35]. Survival of the allogeneic embryo in the uterus depended on the maintenance of immune tolerance at the maternal-fetal interface. The key immune cells that predominantly populated the pregnant uterus were natural killer (NK) cells, which often operated dysfunctionally in patients with miscarriage [36]. In addition, antiphospholipid antibodies (aPL) were linked to recurrent early pregnancy loss (EPL) [37]. All of these aforementioned changes including hyper-activated complement system, NK cell dysfunction, and increased auto-antibodies are prevalent in patients with PCOS, and suggestive of close pathophysiology. Although the causes of PCOS and miscarriage are deemed multi-factorial and cannot be explained in a simple way, the aforementioned findings imply that a kind of PCOS and miscarriage may follow a common pathway and represent different clinical entities. Nevertheless, the detailed mechanisms underlying these diseases are complicated and warrant further investigation.

The use of metformin, an oral biguanide, can ameliorate the hyperinsulinemia and anovulation in PCOS patients [11,30,38], and its safety during pregnancy has been reported in various studies [39]. Our result indicated that the risk of subsequent miscarriage was reduced by about 1/4 for the PCOS patients who underwent metformin treatment compared with those who did not, which was similar to previous studies which reported continuous use of metformin during pregnancy significantly reduced the rate of miscarriage in women with PCOS [39,40]. The underlying mechanisms of PCOS-related miscarriages are still not fully understood; however, tonic LH, hyperinsulinemia, hyperandrogenism, low glycodelin levels in endometrium, hypofibrinolysis mediated by increased plasminogen activator inhibitor (PAI) activity could be involved either alone or in combination in the pathogenesis [39,40]. Recent experimental data showed that PCOS patients had significantly lower serum glycodelin and insulin growth factor binding protein 1 (IGFBP-1) concentrations during the first trimester of pregnancy, suggesting a deficient endometrial environment for implantation and maintenance of pregnancy [40]. It was postulated that a possible mechanism of preventing early miscarriages is the efficacy of metformin in increasing glycodelin in endometrium, which is an adhesive glycoprotein necessary for implantation [39]. Previous research also demonstrated that metformin improves several surrogate markers of endometrial receptivity, blood flow, and ovarian vascularization in PCOS patients [40]. Metformin might help to deal with these complex risk factors of miscarriages [39]. In agreement with other investigations [39,40], our results will further reassure physicians that metformin administration could have a beneficial role in reducing miscarriage risk.

Obesity is a significant risk factor for miscarriage [22]. A possible explanation for the observation of lower miscarriage rate in PCOS patients using metformin is that metformin can improve hyperandrogenism-induced obesity, thus reducing the risk of miscarriage. When used in patients complicated with obesity or metabolic syndrome, metformin can serve as an insulin sensitizer to reduce androgen levels and water retention, resulting in body weight loss. However, the effect of obesity on miscarriage could not be detected in the current study due to a lack of some demographic data such as individual height and body weight.

In contrast to previous studies, the strengths of the nationwide population-based study lay in a large sample size, sound sampling method, and stricter selection criteria for women with the diagnoses of PCOS or miscarriage. A major advantage of the study was the sample, which was collected from the database of a general survey of a national population rather than purposive sampling in some of previous studies. Therefore, the study result is robust because of a relatively large sample size and minimization of potential selection bias resulting from the sampling process. For all of the enrolled women, the diagnoses of PCOS and miscarriage were established based on the objective findings of gynecologic ultrasonography or results of blood tests [2] rather than personal subjective judgment of physicians. All bias originating from the investigator and selection process were eliminated as possible by the database, sample, and criteria we used.

In the current study, the overall miscarriage rate in the whole population was 10.1% (315 + 651 / 7389 + 1275 + 315 + 651). The overall “no-miscarriage” rate in the whole population was 89.9% (7389 + 1275 / 7389 + 1275 + 315 + 651), a bit higher than those reported in the literature. Possible explanations for the observation are that a part of miscarriage is under-reported in the NHI system, and that a part of miscarriage is managed outside the NHI system. On the other hand, the overall miscarriage rate (10.1%) in the whole population (PCOS and non-PCOS women) is lower than those (22/145 = 15.2% and 209/1018 = 20.5%) reported in the previous two studies focusing on women undergoing ART [27,29]. Since ART is indicated and often performed for women with individual factors including old age, poor endometrial condition, ovarian dysfunction, and distorted tubal or uterine anatomy, it is postulated that individual factors themselves in women undergoing ART are subject to infertility, early pregnancy loss and poor reproductive outcomes, accounting for the results of higher miscarriage rates.

Several inherent limitations existed in our study. The first was related to a lack of some demographic and personal data in the NHIRD, such as height and body weight, social and marital status, smoking habits, alcohol, and self-paid medicine use. Therefore, it was not able to inspect the effects of the factors mentioned above. Because individual’s height and body weight were not recorded in the NHIRD of Taiwan, they were unavailable for further analyses. Second, the sole use of ICD9 codes cannot entirely reflect the real clinical condition. Due to the uncertainty of defining diseases from administrative records, pelvic ultrasonography, blood tests and clinical records were all identified to verify the diagnoses. Moreover, administrative records were uploaded to the NHI system mainly for the sake of reimbursement. The inconsistency in the sequentially gathered data might affect the results of the study. Furthermore, the information of duration and dosage about metformin usage was absent, which was an inevitable and congenital limitation of the nationwide database. Finally, our study could not investigate the effect of race on subsequent miscarriage because of the racial homogeneity in Taiwan.

## 5. Conclusions

The result of the nationwide population-based study has demonstrated that pre-pregnancy PCOS is an independent and significant risk factor for subsequent miscarriage of pregnant women. Nevertheless, the risk of subsequent miscarriage is reduced by about 1/4 for the PCOS patients who undergo metformin treatment compared with those who do not. Relevant information and suggestions should be offered to at-risk women to facilitate specialty referral and early management. For affected women, counselling and management can be arranged in response to potentially physical effects and psychological impacts.

## Figures and Tables

**Table 1:** The characteristics of pregnant women with and without prior PCOS.

	Case Group		Control Group		Statistics		
	Women with Prior PCOS		Women without Prior PCOS				
	(*n* = 1926)		(*n* = 7704)				
	*N*	%	*N*	%	OR	[95% CI]	*p*-Value
**Age at PCOS diagnosis (y/o)**	27.31 ± 5.06						
15–25	701	36.40					
26–35	1116	57.94					
36–45	109	5.66					
**Age at first pregnancy (y/o)**	30.03 ± 4.66		27.33 ± 4.56				<0.0001 ***
**Occupation**							<0.0001 ***
White collar	1188	61.68	4318				
Blue collar	326	16.93	1415	18.37			
Retired and others	412	21.39	1971	25.58			
**Urbanization**							0.0005 **
Urban	1319	68.48	4935	64.06			
Suburban	492	25.55	2311	30.00			
Rural	115	5.97	458	5.94			
**Economic status (insurable wage)**							<0.0001 ***
≥40,000 NTD	410	21.29	1195	15.51			
20,000–40,000 NTD	833	43.25	3351	43.50			
<20,000 NTD 407 21.13 1836 23.83							
Retired and others	276	14.33	1322	17.16			
**Co-morbidities**							
Diabetes mellitus	83	4.31	101	1.31	3.39	[2.52–4.55]	<0.0001 ***
Hypertension	33	1.71	112	1.45	1.18	[0.80–1.75]	0.4027
Dyslipidemia	89	4.62	223	2.89	1.63	[1.26–2.09]	0.0001 **
Ischemic heart disease	25	1.30	111	1.44	0.90	[0.58–1.39]	0.6348
Cerebrovascular disease	14	0.73	81	1.05	0.69	[0.39–1.22]	0.1975
Chronic pulmonary disease	293	15.21	1062	13.79	1.12	[0.98–1.29]	0.1070
Autoimmune disease	124	6.44	443	5.75	1.13	[0.92–1.39]	0.2513

Data are expressed as the number (%) or mean ± standard deviation, as appropriate. ** *p* < 0.001, *** *p* < 0.0001, by chi-square test or student t test, as appropriate.

**Table 2:** The risk analysis of subsequent miscarriage for pregnant women with prior PCOS.

	No Miscarriage		MISCARRIAGE		Statistics	
	*N*	%	*N*	%	Crude OR ^a^	Adjusted OR ^b^
					[95% CI]	[95% CI]
**Group**						
Control group: (*n* = 7704)	7389	95.91	315	4.09	Reference	Reference
women without PCOS						
Case group: (*n* = 1926)	1275	66.20	651	33.80	11.98 * [10.34–13.87]	11.97 * [10.27–13.95]
women with PCOS						
*Sub-group in case group*						
*No Metformin sub-group*	1146	65.75	597	34.25	12.22 * [10.52–14.20]	12.13 * [10.38–14.18]
(*n* = 1743)						
*Metformin sub-group*	129	70.49	54	29.51	9.82 * [7.01–13.76]	9.53 * [6.69–13.57]
(*n* = 183)						

* *p* < 0.0001; ^a^ Odds ratio (OR) and 95% confidence intervals [95% CI] are calculated by logistic regression analysis, as compared to the reference group. ^b^ Adjusted for age at first pregnancy, occupation, urbanization, economic status, and co-morbidities.

## Data Availability

Regarding data availability, the data utilized in this study cannot be made available in the manuscript, the supplemental files, or in a public repository due to the Taiwan Personal Information Protection Act, but the data from the National Health Insurance Research Database is available for application from the National Health Research Institutes in Taiwan for researchers who meet the criteria for data access. Further information regarding data access is available at the National Health Research Institutes website (http://nhird.nhri.org.tw) or by email to nhird@nhri.org.tw.
